# Exploring Combinations of Different Color and Facial Expression Stimuli for Gaze-Independent BCIs

**DOI:** 10.3389/fncom.2016.00005

**Published:** 2016-01-29

**Authors:** Long Chen, Jing Jin, Ian Daly, Yu Zhang, Xingyu Wang, Andrzej Cichocki

**Affiliations:** ^1^Key Laboratory of Advanced Control and Optimization for Chemical Processes, Ministry of Education, East China University of Science and TechnologyShanghai, China; ^2^Brain Embodiment Lab, School of Systems Engineering, University of ReadingReading, UK; ^3^Riken Brain Science InstituteWako-shi, Japan; ^4^Systems Research Institute of Polish Academy of SciencesWarsaw, Poland; ^5^Skolkovo Institute of Science and TechnologyMoscow, Russia

**Keywords:** event-related potentials, brain-computer interface (BCI), dummy face, fusion stimuli, gaze-independent, facial expression

## Abstract

**Background:** Some studies have proven that a conventional visual brain computer interface (BCI) based on overt attention cannot be used effectively when eye movement control is not possible. To solve this problem, a novel visual-based BCI system based on covert attention and feature attention has been proposed and was called the gaze-independent BCI. Color and shape difference between stimuli and backgrounds have generally been used in examples of gaze-independent BCIs. Recently, a new paradigm based on facial expression changes has been presented, and obtained high performance. However, some facial expressions were so similar that users couldn't tell them apart, especially when they were presented at the same position in a rapid serial visual presentation (RSVP) paradigm. Consequently, the performance of the BCI is reduced.

**New Method:** In this paper, we combined facial expressions and colors to optimize the stimuli presentation in the gaze-independent BCI. This optimized paradigm was called the colored dummy face pattern. It is suggested that different colors and facial expressions could help users to locate the target and evoke larger event-related potentials (ERPs). In order to evaluate the performance of this new paradigm, two other paradigms were presented, called the gray dummy face pattern and the colored ball pattern.

**Comparison with Existing Method(s):** The key point that determined the value of the colored dummy faces stimuli in BCI systems was whether the dummy face stimuli could obtain higher performance than gray faces or colored balls stimuli. Ten healthy participants (seven male, aged 21–26 years, mean 24.5 ± 1.25) participated in our experiment. Online and offline results of four different paradigms were obtained and comparatively analyzed.

**Results:** The results showed that the colored dummy face pattern could evoke higher P300 and N400 ERP amplitudes, compared with the gray dummy face pattern and the colored ball pattern. Online results showed that the colored dummy face pattern had a significant advantage in terms of classification accuracy (*p* < 0.05) and information transfer rate (*p* < 0.05) compared to the other two patterns.

**Conclusions:** The stimuli used in the colored dummy face paradigm combined color and facial expressions. This had a significant advantage in terms of the evoked P300 and N400 amplitudes and resulted in high classification accuracies and information transfer rates. It was compared with colored ball and gray dummy face stimuli.

## Introduction

A brain-computer interface is designed to establish a communication channel between a human and external devices, without the help of peripheral nerves and muscle tissue (Wolpaw et al., 2002; Neuper et al., 2006; Allison et al., 2007; Mak and Wolpaw, 2009; Treder et al., 2011; Rodríguez-Bermúdez et al., 2013). Event-related potential (ERP)-based BCIs are able to obtain high classification accuracy and information transfer rates. Consequently, they are one of the most widely used BCI systems. ERP-based BCIs are used to control external devices such as wheel-chairs, spelling devices, and computers (Lécuyer et al., 2008; Cecotti, 2011; Li et al., 2013; Yin et al., 2013).

The N200, P300, and N400 ERPs are most frequently used in ERP-based BCIs. The P300 component is a positive potential, observed at central and parietal electrode sites about 300–400 ms after stimulus onset, which can be observed during an oddball paradigm (Polich, 2007; Acqualagna and Blankertz, 2013). The N200 and N400 components are negative potentials, which can be observed approximately 200–300 and 400–700 ms after stimulus onset (Polich, 2007).

A P300 BCI is a typical example of a BCI system based on visual, audio, or tactile stimuli (Hill et al., 2004; Fazel-Rezai, 2007; Kim et al., 2011; Mak et al., 2011; Jin et al., 2014a; Kaufmann et al., 2014). The first P300-based BCI system was presented by Farwell and Dochin using a 6 × 6 matrix of letters (Farwell and Donchin, 1988). This stimulus matrix was a mental typewriter, which consisted of symbols laid out in six rows and six columns. The user focused on one of symbols while the rows and columns were highlighted in a random order. The symbol the user focusses on (the target) could be identified based on the classification result of the ERPs. However, the information transfer rate and classification accuracy of the system was not high enough for practical applications. Many studies had been conducted to attempt to improve classification accuracies and information transfer rates of the P300 speller (Donchin et al., 2000; Guan et al., 2004; Townsend et al., 2010; Bin et al., 2011; Jin et al., 2011; Zhang et al., 2011).

One of the main goals of BCI is to help people who have lost the ability to communicate or control external devices. Most of the visual-based BCI systems use a matrix like Forwell and Donchin's system (Farwell and Donchin, 1988). However, recent studies have shown that the matrix-based speller does not work well for individuals who are not able to control their gaze (Brunner et al., 2010; Treder and Blankertz, 2010; Frenzel et al., 2011). In these patterns, the absence of early occipital components reduced classification performance (Brunner et al., 2010). It has been proved that these components are modulated by overt attention and contribute to classification performance in BCI systems (Shishkin et al., 2009; Bianchi et al., 2010; Brunner et al., 2010; Frenzel et al., 2011). Furthermore, Frenzel et al's research suggested that the occipital N200 component mainly indexed the locus of eye gaze and that the P300 mainly indexed the locus of attention (Frenzel et al., 2011).

In view of the above problems, researchers have made efforts to develop BCI systems which are independent of eye gaze. A possible solution is to use non-visual stimuli such as auditory or tactile stimuli (Klobassa et al., 2009; Kübler et al., 2009; Brouwer and van Erp, 2010; Chreuder et al., 2011; Schreuder et al., 2011; Lopez-Gordo et al., 2012; Thurlings et al., 2012). Additionally, for visual-based BCI systems, some new areas of research were developed (Marchetti et al., 2013; An et al., 2014; Lesenfants et al., 2014). For example, Treder et al. presented the first study on gaze independent BCIs in 2010 (Treder and Blankertz, 2010). Following on from this work they optimized the gaze-independent pattern by using non-spatial feature attention and facilitating spatial covert attention in 2011 (Treder et al., 2011).

Schaeff et al. reported a gaze-independent paradigm with motion VEPs in 2012 (Schaeff et al., 2012). Acqualagna et al. presented a novel gaze-independent paradigm called the rapid serial visual presentation (RSVP) paradigm in 2010 (Acqualagna et al., 2010). In this paradigm, all the symbols were presented one by one in a serial manner and in the center of the display. The classification accuracy of these presented paradigms was acceptable and could work for individuals who could not control their gaze. However, the classification accuracies and information transfer rates of these gaze-independent BCIs should be improved further for practical applications. The common stimuli used in gaze-independent paradigms were letters, numbers, and polygons, which were used to evoke false positive ERPs in the non-target trials.

It had been proven that face stimuli could be used to obtain high BCI performance (Jin et al., 2012; Kaufmann et al., 2012; Zhang et al., 2012). Facial expressions on dummy faces could evoke strong ERPs (Curran and Hancock, 2007; Chen et al., 2014; Jin et al., 2015). Additionally, the use of different facial expressions has been shown to produce different ERP amplitudes during a BCI control experiment. In our study, the stimuli were colored ball and dummy faces, which were only composed of simple lines and arcs. Meanwhile, dummy faces could show a person's facial expressions and are easily edited without copyright infringement problems. That is, every face was a cartoon face. The primary goal of this study was to survey whether the stimuli, which combined different facial expression and colors, could obtain higher performance compared with the traditional gaze independent pattern.

Different colors and facial expressions were used to help participants to locate the target stimulus, resulting in enlarged evoked ERPs when the participant was focusing on the target. Furthermore, different facial expressions were used to decrease the repetition effects in evoking the ERPs (Jin et al., 2014b). To evaluate the validity of the colored dummy face pattern in increasing information transfer rates, improving classification accuracies, and evoking ERPs, three different paradigms were presented, which were called the colored dummy face pattern, the gray dummy face pattern, and the colored ball pattern. The colored ball pattern was a gaze-independent paradigm using different colors; the gray dummy face pattern was a gaze-independent paradigm using different facial expressions; finally, the colored dummy face pattern was a gaze-independent paradigm that combined the different colors and facial expressions.

## Materials and methods

### Participants

Ten healthy individuals (seven male, aged 21–26 years, mean 24.5 ± 1.25) participated in this study, and were marked as S1, S2, S3, S4, S5, S6, S7, S8, S9, and S10. The 10 participants signed a written consent form prior to this experiment and were paid for their participation. The local ethics committee approved the consent form and experimental procedure before any individuals participated. The native language of all the participants was Mandarin Chinese.

### Stimuli and procedure

**Figure 1:**
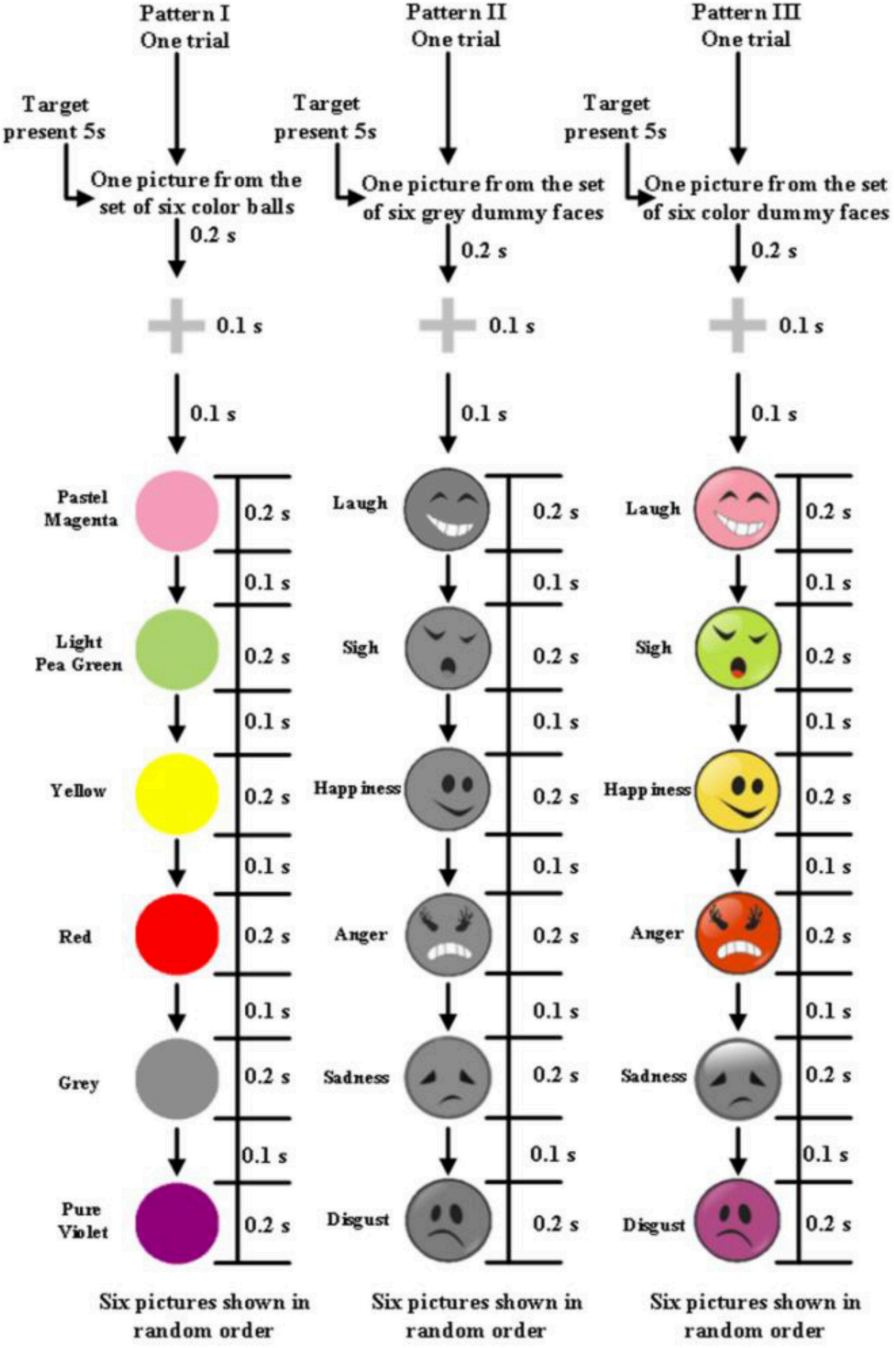
**One trial of the experiment**. Pattern I is the colored ball paradigm, pattern II is the gray dummy face paradigm, and pattern III is the colored dummy face paradigm.

After being prepared for EEG recording, the participants were seated in a comfortable chair 70 ± 3 cm in front of a standard 24 inch LED monitor (60 Hz refresh rate, 1920 × 1080 screen resolution) in a shielded room. The stimuli were presented in the middle of the computer screen. During data acquisition, participants were asked to relax and avoid unnecessary movement. There were three experimental paradigms, the colored ball paradigm (Pattern I), the gray dummy face paradigm (Pattern II), and the color dummy face paradigm (Pattern III). Every dummy face paradigm included six different cartoon face stimuli, which were taken from the internet and modified with Photoshop 7.0. These face stimuli encode six facial expressions, which could be facially encoded laughter, sighing, happiness, anger, sadness, or disgust. These facial stimuli had the same size and lighting. The stimuli used in these three paradigms are shown in Figure 1. Every dummy face picture consisted of simple lines and arcs.

**Figure 2:**
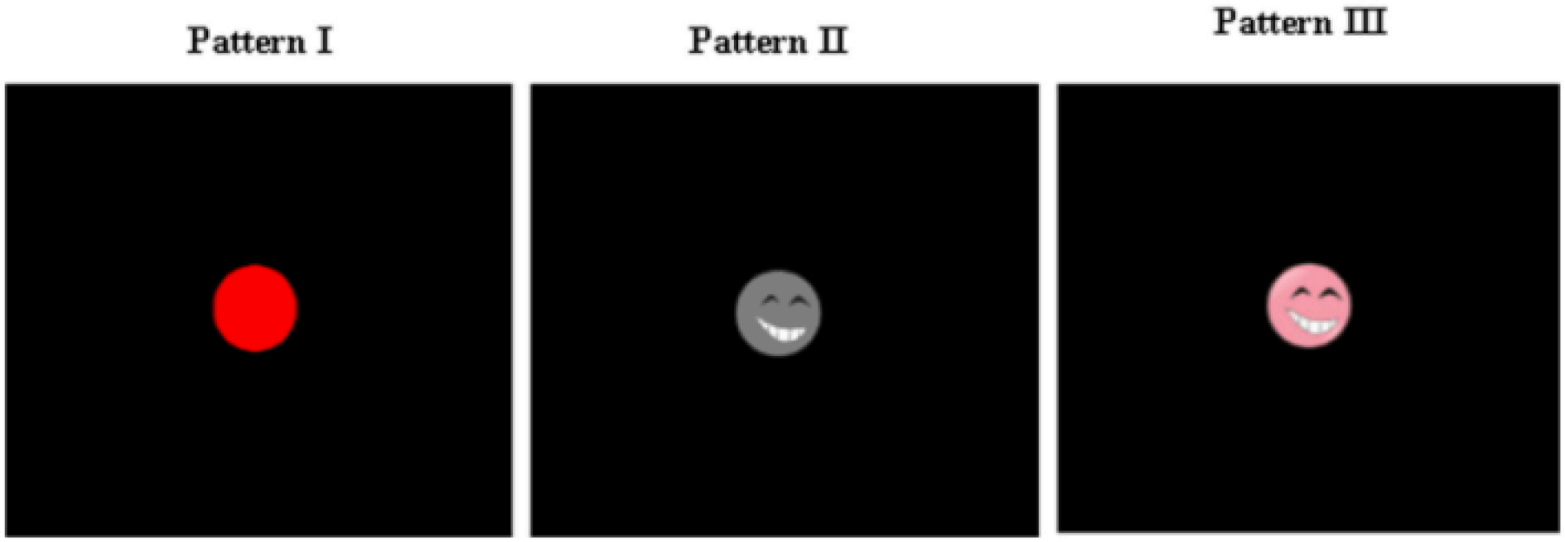
**The stimulus display**. The stimulus is shown in a layout that is independent of eye gaze.

Every picture as a stimulus was shown in the middle of a computer screen (Figure 2). The serial number of each picture (including target and non-target) was shown at the top of the screen. Only three conditions differed between the stimuli images. Every condition contained six pictures (Figure 1). The flash stimulus on time was 200 ms, and the off time was 100 ms (Figure 1). After the stimuli off time there was nothing in the screen. The stimuli of three paradigms were illustrated in Figure 2.

### Experiment setup, offline, and online protocols

**Figure 3:**
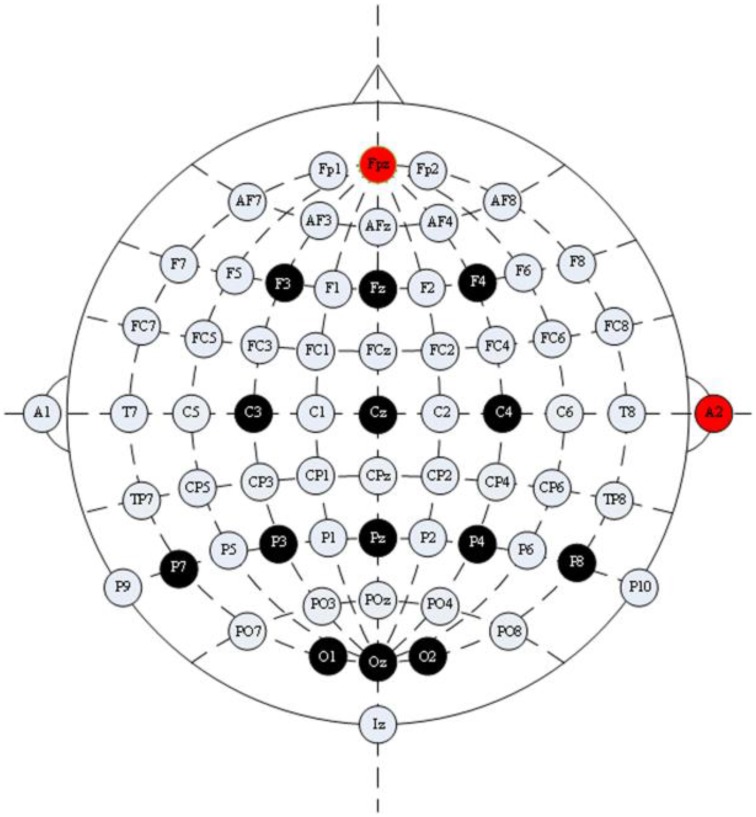
**Configuration of electrode positions**. The electrode positions used in our experiment were F3, F4, Fz, C3, C4, Cz, P7, P3, Pz, P4, P8, O1, O2, and Oz; Fpz was used as the ground electrode position; A2 was used as the reference electrode position.

EEG signals were recorded with a g.USBamp and a g.EEGcap (Guger Technologies, Graz, Austria) with a sensitivity of 100 μV, band pass filtered between 0.1 and 30Hz, and sampled at 256Hz. We recorded from 14 EEG electrode positions based on the extended International 10–20 system (Figure 3). These electrodes were Cz, Pz, Oz, Fz, F3, F4, C3, C4, P3, P4, P7, P8, O1, and O2. The right mastoid electrode was used as the reference and the front electrode (FPz) was used as the ground. Data were recorded and analyzed by using the ECUST BCI platform software package which was developed by East China University of Science and Technology (Jin et al., 2011).

**Figure 4:**
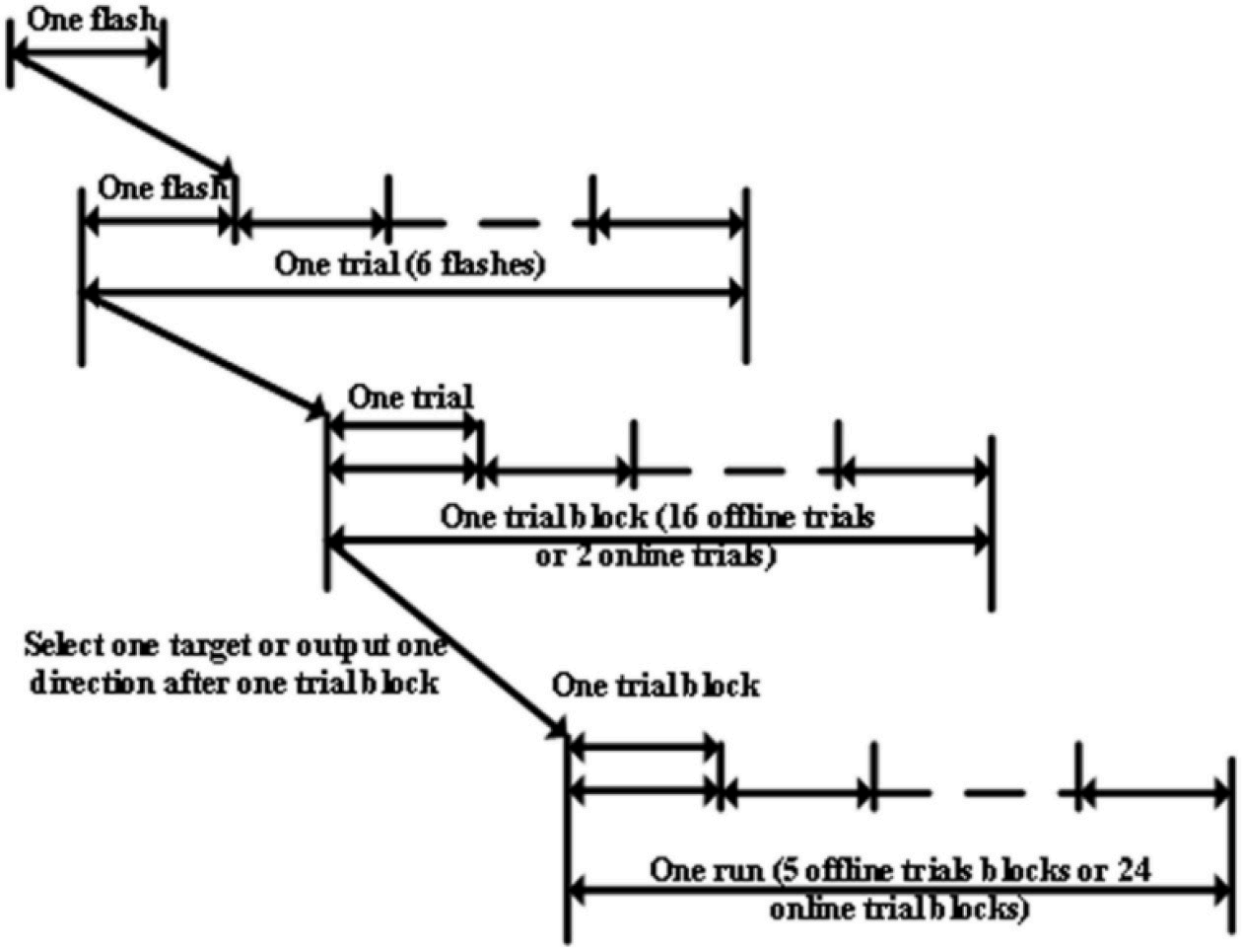
**One run of the experiment for online and offline experiments**.

In this paper, the term “flash” referred to each individual event. A single character flash pattern was used here. In each trial, each ball, or dummy face, was flashed once. In other words, each trial included six flashes. All patterns had 200 ms of flashes followed by a 100 ms delay, and each trial lasted 1.8 s with one target and five non-targets (see Figure 1). A trial block referred to a group of trials with the same target. During the offline experiment, there were 16 trials per trial block and each run consisted of five trial blocks, each of which involved a different target. Participants had a 2 min break after each offline run. During the online experiment participants attempted to identify 24 targets (see Figure 4).

There were three conditions, which were presented to each participant in random order. For each condition, participants first took part in three offline runs. Participants had 2 min rest between each offline run (Figure 4). After all offline runs, participants were asked to attempt to identify 24 targets for each pattern in the online experiment. Feedback and target selection time was 5 s before each trial block. Participants had 2 min rest before starting the online task for each condition. The target cue (a dummy face or a colored ball) was shown in the middle of the screen for 2 s before each run. Participants were instructed to focus on, and count, appearances of this cue during both the online and offline experiments. The feedback, which was obtained during the online experiments, was shown at the top of the screen.

### Feature extraction procedure

A third-order Butterworth band pass filter was used to filter the EEG between 0.1 and 30 Hz. The EEG was then down-sampled from 256 to 51 Hz by selecting every fifth sample from the filtered EEG. A single flash, which lasted 800 ms, was extracted from the data. For the offline data, Windsorizing was used to remove the electrooculogram (EOG). The 10th percentile and the 90th percentile were computed for the samples from each electrode. Amplitude values lying below the 10th percentile or above the 90th percentile were, respectively, replaced by the 10th percentile or 90th percentile (Hoffmann et al., 2008).

### Classification scheme

Bayesian linear discriminant analysis (BLDA) is an extension of Fisher's linear discriminant analysis (FLDA) that avoids over fitting and possibly noisy datasets. The detail of the algorithm can be found in Hoffmann et al. (2008). By using a Bayesian analysis, the degree of regularization can be estimated automatically and quickly from the training data (Hoffmann et al., 2008). Data acquired from the offline experiment was used to train the classifier using the BLDA classifier to obtain the classifier model. This model is then used in the online system.

### Raw bit rate and practical bit rate

In this paper, we used a bit rate calculation method called raw bit rate (RBR), which was calculated via

(1) $$\begin{array}{l} B=\left\{\log_{2}N+P\log_{2}P+\left(1-P\right)\log_{2}\left[\left(1-P\right)∕\left(N-1\right)\right]\right\}\times T \end{array}$$

where *P* denotes the classification accuracy and *N* denotes the number of target every trial. *N* was equal to six in our experiment. *T* denotes the completion time of the target selection task. Bit rate is an objective measure for measuring BCI performance and for comparing different BCIs (Wolpaw et al., 2002). RBR is calculated without selection time as defined in Wolpaw et al. (2002).

Practical bit rate (PBR) may be used to estimate the speed of the system in a real-world setting. PBR incorporates the fact that every error requires two additional selections to correct the error. Thus, selecting the wrong character is followed by a backspace and the selection of the correct character. The PRB is calculated as RBR ^*^ (1 – 2 ^*^ P1), where RBR is the raw bit rate and P is the online error rate of the system (Townsend et al., 2010). If *P* > 50%, PRB is zero. The PBR also incorporates the time between selections (4 s).

## Results

### Offline analysis

In this paper, electrode P7 was selected to measure the amplitude of N200 difference (Hong et al., 2009); electrode Cz was selected to measure the amplitude of P300 (Treder et al., 2011); and electrode Fz was selected to measure the amplitude of the N400 (Jin et al., 2014a).

**Figure 5:**
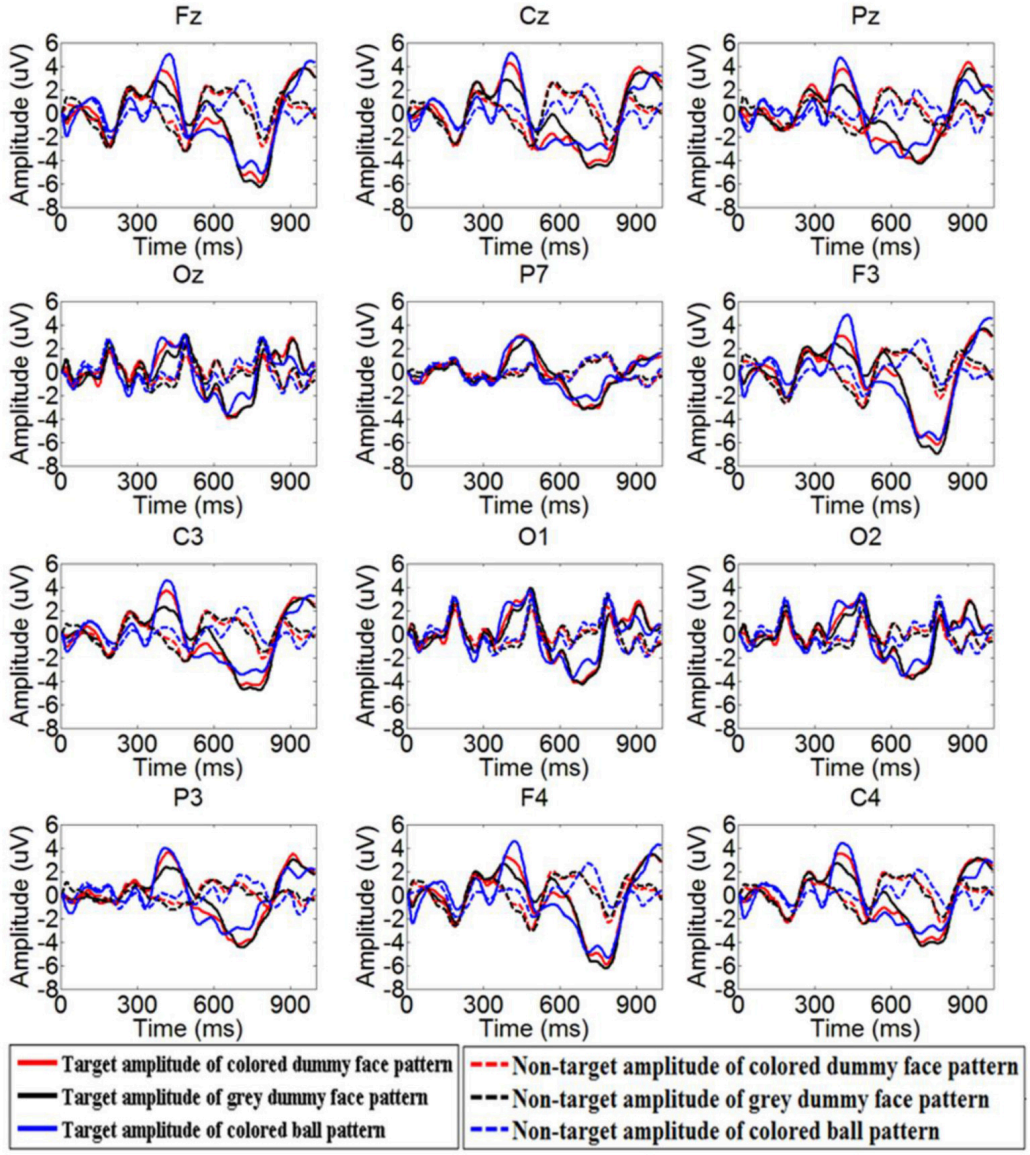
**Grand averaged ERPs of target flashes across all participants over 12 electrode sites**. The red line, black line, and green line indicate the target amplitude of the colored dummy face pattern, gray dummy face pattern, and colored ball pattern, respectively.

The mean latency and amplitude of ERPs from all 10 participants is shown in Table 1. Figure 5 shows the grand averaged amplitudes of target and non-target flashes across all participants over 12 electrode sites for the colored dummy face pattern, the gray dummy face pattern, and the colored ball pattern. Specifically, frontal and central channels contain an early negative ERP at around 250 ms (N200), followed by a high positive potential at around 350 ms (P300), and then a larger negative ERP at around 700 ms (N400).

**Table 1:** **Averaged peak values and averaged latency of N200 at P7, P300 at Cz, and N400 at Fz**.

**ERP**	**Electrodes**	**Amplitude (**μ**V)**			**Latency (ms)**		
		**CDF-P**	**GDF-P**	**CB-P**	**CDF-P**	**GDF-P**	**CB-P**
N200	P7	−1.5941	−1.6428	−1.8780	259.22	270.16	248.13
P300	Cz	5.2707	4.1858	5.9231	391.82	376.73	387.91
N400	Fz	−7.1997	−7.5562	−5.4153	693.75	719.14	667.97

“*CDF-P” denotes the colored dummy face pattern, “GDF-P” denotes the gray dummy face pattern, and “CB-P” denotes the colored ball pattern*.

**Figure 6:**
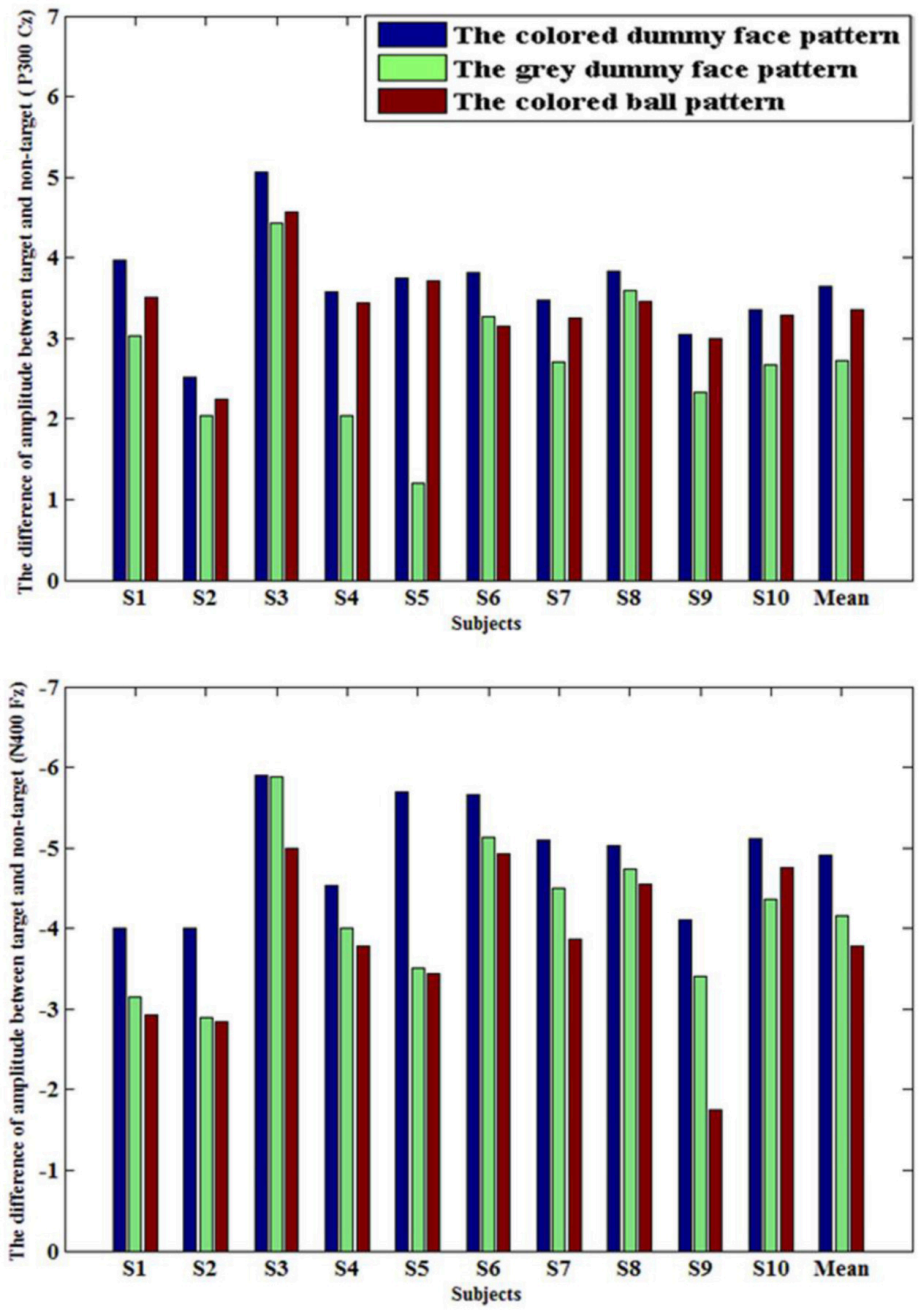
**Upper part:** The amplitude difference of P300 between target and non-target ERP amplitudes at electrode Cz across all 10 participants (μV); **Lower part:** The amplitude difference of N400 between target and non-target ERP amplitudes at electrode Fz across all 10 participants (μV).

Figure 6 shows the amplitude differences between target and non-target ERPs at Cz (P300, peak point ± 25 ms), and at Fz (N400, peak point ± 25 ms). A one-way repeated measures ANOVA was used to show P300 peak amplitude [*F*_(2, 27)_ = 4.1, *p* = 0.0279], and N400 peak amplitude [*F*_(2, 27)_ = 3.9, *p*= 0.0324) among three patterns. It was shown that the colored dummy face pattern evoked a significantly higher P300 ERP (*p* < 0.05) and N400 ERP (*p* < 0.05) compared to other two patterns.

**Figure 7:**
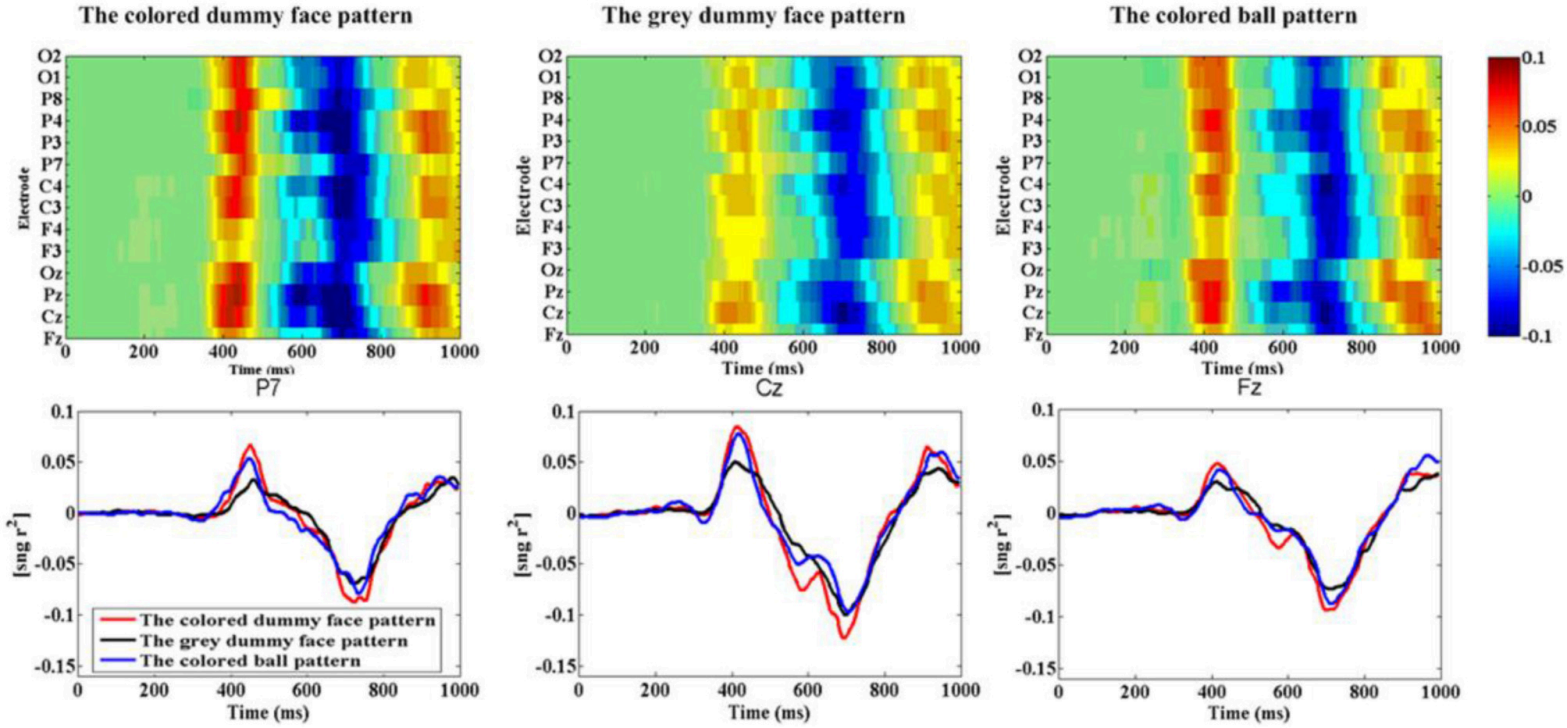
***R*-squared values of ERPs**. **(A)** *R*-squared values of ERPs from the three paradigms between 1 and 1000 ms averaged from participants 1–10 at sites Fz, Cz, Pz, Oz, F3, F4, C3, C4, P7, P3, P4, P8, O1, and O2. **(B)** The *r*-squared values at P7, Cz, and Fz.

Figure 7 shows the grand average *r*-squared values of ERPs across all 10 participants at site Fz, Cz, Pz, Oz, F3, F4, C3, C4, P7, P3, P4, P8, O1, and O2. A one-way repeated measures ANOVA was used to show the *r*-squared value difference of the N200 ERP across all 10 participants. It was significant at electrode P7 [F_(2, 27)_ = 0.12, *p* = 0.91] for the N200 (peak point ± 25 ms), at Cz [*F*_(2, 27)_ = 3.74, *p* = 0.0368] for the P300 (peak point ± 25 ms), and at Fz [F_(2, 27)_ = 3.38, *p* = 0.049] for the N400 (peak point ± 25 ms). It was shown that the colored dummy face pattern obtained significantly higher *r*-squared values during the P300 ERP (*p* < 0.05) and the N400 ERP (*p* < 0.05) compared to the other two patterns.

**Figure 8:**
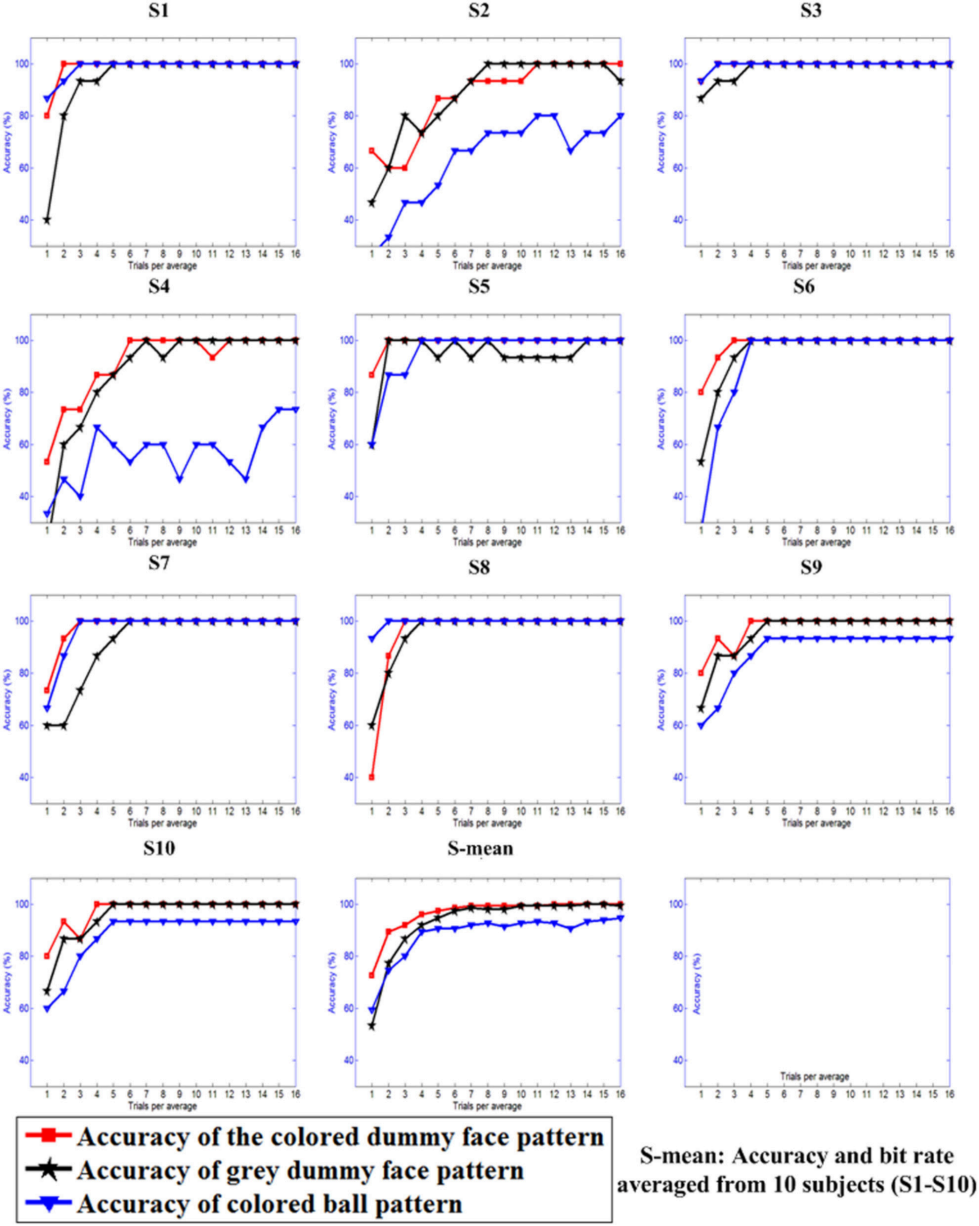
**Offline classification accuracies with differing numbers of trials used for constructing the averaged ERPs**.

Figure 8 shows the offline classification accuracies of the three patterns when differing numbers of trials (1–16) were used to construct the averaged ERP.

**Figure 9:**
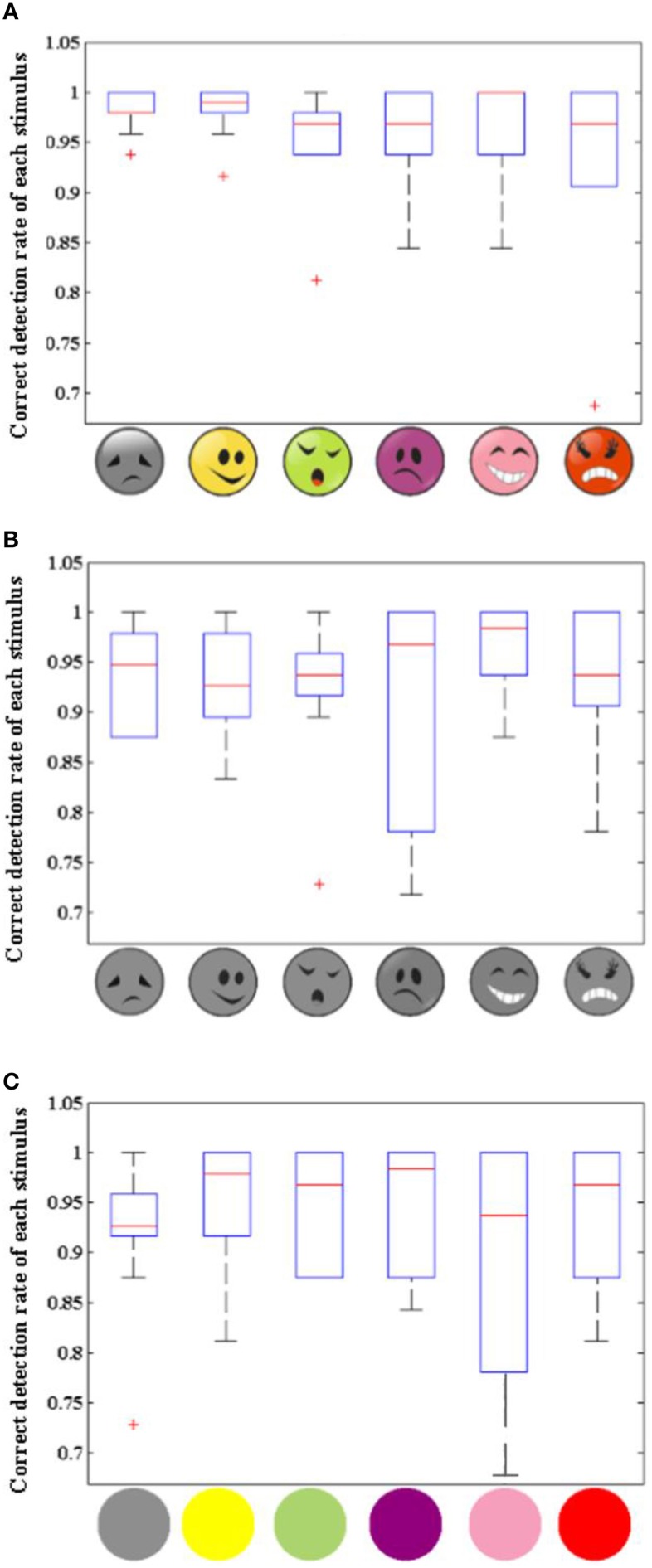
**Boxplot of correct detection rate of each stimulus for 10 participants**. Panels **(A)**, **(B)**, and **(C)** are the Boxplots of correct detection rate of each stimulus across all 10 participants for the colored dummy face pattern **(A)**, the gray dummy face pattern **(B)**, and the colored ball pattern **(C)**, respectively.

Figure 9 shows three boxplots, which illustrate the distribution of correct detection rates of each stimulus across all 10 participants for the three patterns. Correct detection rate of a stimulus shows the rate of correct classification for one stimulus. It indicates that the classification accuracy of the six stimuli in the colored dummy face pattern was the most stable when compared to the other two patterns.

### Online results

Table 2 shows the online classification accuracies and information transfer rates with an adaptive strategy (Jin et al., 2011). A one-way repeated measures ANOVA was used to show the difference in classification accuracies [*F*_(2, 27)_ = 4.27, *p* = 0.0245], RBRs [*F*_(2, 27)_ = 4.17, *p* = 0.0264], and PBR [*F*_(2, 27)_ = 4.63, *p* = 0.0186] across three patterns. It shows that the colored dummy face pattern achieved significantly higher classification accuracies (*p* < 0.05), RBRs (*p* < 0.05), and PRB (*p* < 0.05), than the gray dummy face pattern and the colored ball pattern.

**Table 2:** **Classification accuracy, raw bit rate, and practical bit rates achieved during online experiments**.

		**S1**	**S2**	**S3**	**S4**	**S5**	**S6**	**S7**	**S8**	**S9**	**S10**	**Average**
Acc (%)	CDF-P	100	83.3	100	79.2	100	91.7	100	95.8	95.8	100	95.0 ± 7.6
	GDF-P	95.8	79.1	100	62.5	91.7	83.3	83.3	100	95.8	70.8	86.2 ± 12.7
	CB-P	91.7	54.2	91.7	45.8	95.8	87.5	75.0	79.2	79.2	83.3	78.3 ± 16.4
RBR	CDF-P	37.6	20.3	39.0	19.1	39.8	27.3	36.3	33.8	28.9	35.7	31.8 ± 7.5
	GDF-P	32.6	17.9	39.0	10.7	30.4	21.4	21.7	39.0	28.9	14.3	25.6 ± 9.9
	CB-P	29.9	6.3	29.3	3.7	31.4	25.0	16.5	17.3	20.6	21.0	20.1 ± 9.5
PBR	CDF-P	17.8	6.4	18.5	5.3	18.8	10.8	17.2	14.7	12.5	16.9	13.9 ± 5.0
	GDF-P	14.1	4.9	18.5	1.3	12.0	6.7	6.9	18.5	12.5	2.8	9.8 ± 6.2
	CB-P	11.8	0.2	11.6	0	13.6	8.9	3.9	4.8	5.7	5.8	6.6 ± 4.7

*In this table, “Acc” refers to classification accuracy, “RBR” refers to raw bit rate, measured in bits/min, and “PBR” refers to practical bit rate, measured in bits/min. “CDF-P” denotes the colored dummy face pattern, “GDF-P” denotes the gray dummy face pattern, and “CB-P” denotes the colored ball pattern*.

## Discussion

The primary goal of this study was to verify that the colored dummy face pattern could increase the distinguishability of stimuli, and improving classification accuracies and information transfer rates. Facial expression stimuli could evoke strong N200, P300, and N400 ERPs, while colored stimuli could also evoke high P300 ERPs, especially in gaze-independent paradigms (Treder et al., 2011; Acqualagna and Blankertz, 2013; Chen et al., 2014; Jin et al., 2015). In this paper, the different colors and face expressions were combined to produce enlarged ERPs. The results show that higher N400 and P300 ERPs were evoked by the colored dummy face pattern, compared with the gray dummy face pattern and the colored ball pattern. The colored dummy face pattern had a significant advantage in terms of the P300 (*p* < 0.05) and N400 (*p* < 0.05) ERP amplitudes (see Figure 6) compared to other two patterns. Furthermore, the colored dummy face pattern had an advantage over other two patterns in terms of *r*-squared values of ERP amplitudes at Cz (*p* < 0.05) for the P300 ERP, and the N400 ERP at Fz *p* < 0.05).

### Classification accuracy and information transfer rate

Classification accuracy and information transfer rate are two important indexes to measure the performance of BCI systems. Online classification accuracies and information transfer rates of the three patterns are shown in Table 2. A one-way repeated ANOVA was used to show the difference in classification accuracy (*F* = 4.27, *p* < 0.05), RBR (*F* = 4.17, *p* < 0.05), PBR (*F* = 4.63, *p* < 0.05) between the three patterns. It showed that the use of the colored dummy face pattern resulted in significantly higher classification accuracies (*p* < 0.05), RBRs (*p* < 0.05), and PBRs (*p* < 0.05), compared with the gray dummy face pattern and the colored ball pattern. The mean classification accuracy of the colored dummy face pattern was 8.8% higher than that of the gray dummy face pattern, while the mean RBR of the colored dummy face pattern was 6.2 bits min^−1^ higher than that of the gray dummy face pattern. The mean classification accuracy and RBR of the colored dummy face pattern are 16.7% and 11.7 bit min^−1^ higher than those of the colored ball pattern.

### Potential advantage for users

The system is a kind of gaze-independent BCI, which is based on RSVP, which could be used by individuals who completely or partially lost their ability to control their eye gaze. Figure 9 shows that the stimuli used in the colored dummy face pattern is more stable for all participants compared to the other two patterns, which shows the advantage of the colored dummy face pattern for practical applications.

## Conclusions

A colored dummy face paradigm for visual attention-based BCIs was presented. The stimuli used in this pattern combined colors and facial expressions, which lead to high classification accuracies during RSVP. It had a significant advantage in terms of the evoked P300 and N400 amplitudes. It was also able to obtain high classification accuracies and information transfer rates, compared with the color change paradigm and the facial expression paradigm. In the future we will further verify the performance of this paradigm with patients.

## Author contributions

LC and JJ had designed and finished the experiment. ID, YZ, XW, and AC gave guidances. LC completed the manuscript. JJ and ID modified manuscript. XW provided the required experimental funds.

## Funding

This work was supported in part by the Grant National Natural Science Foundation of China, under Grant Nos. 61573142, 61203127, 91420302, and 61305028. This work was also supported by the Fundamental Research Funds for the Central Universities (WG1414005, WH1314023, and WH1516018) and Shanghai Chenguang Program under Grant 14CG31.

### Conflict of interest statement

The authors declare that the research was conducted in the absence of any commercial or financial relationships that could be construed as a potential conflict of interest.
